# Water‐filtered infrared A radiation hyperthermia combined with immunotherapy for advanced gastrointestinal tumours

**DOI:** 10.1002/cam4.70024

**Published:** 2024-07-24

**Authors:** Pengyuan Liu, Jing Wu, Liting Chen, Zhenhai Wu, Yufei Wu, Ganlu Zhang, Bingqi Yu, Beibei Zhang, Nan Wei, Jinan Shi, Chuanfeng Zhang, Lan Lei, Shuhuan Yu, Jianjun Lai, Zhen Guo, Yuli Zheng, Zhao Jing, Hao Jiang, Tingxiang Wang, Jueyi Zhou, Yajun Wu, Chuan Sun, Jie Shen, Jian Zhang, Zhibing Wu

**Affiliations:** ^1^ Department of Oncology, Zhejiang Hospital Hangzhou China; ^2^ ACS (International) School of Singapore Singapore; ^3^ Department of Oncology Lishui People's Hospital Lishui China; ^4^ TCM Dispensary, Zhejiang Hospital Hangzhou China; ^5^ Geriatrics Institute of Zhejiang Province Department of Geriatrics, Zhejiang Hospital Hangzhou China; ^6^ Department of Medical Oncology, The First Affiliated Hospital Zhejiang University School of Medicine Hangzhou China; ^7^ Department of Gastrointestinal Surgery, The First Affiliated Hospital Zhejiang University School of Medicine Hangzhou China; ^8^ Department of Radiation Oncology, Affiliated Zhejiang Hospital Zhejiang University School of Medicine Hangzhou China; ^9^ Cancer Center Zhejiang University Hangzhou China

## Abstract

**Introduction:**

This study aimed to validate the effectiveness, safety and feasibility of water‐filtered infrared A radiation (WIRA) whole‐body hyperthermia combined with immune checkpoint inhibitor (ICI) therapy (HIT) and evaluate the real‐world clinical application prospects.

**Methods:**

This open‐label single‐arm phase 2 clinical trial (NCT06022692) aimed to enrol advanced gastrointestinal tumour (GIT) patients with the MSS/pMMR phenotype. The patients were treated with whole‐body hyperthermia on Days 1 and 8 of each HIT cycle along with administration of tislelizumab on Day 2.

**Results:**

Between 1 June 2020 and 31 May 2022, 18 patients were enrolled in the study, including those with gastric cancer (*n* = 6), colon cancer (*n* = 7), rectal cancer (*n* = 3) and appendiceal cancer (*n* = 2). As of 19 May 2023, 17 of the 18 patients had died, including 14 deaths caused by tumour progression and three deaths caused by diseases other than cancer, while one patient was still undergoing follow‐up. In terms of efficacy, the median DCR was 55.6%, while the median PFS and OS were 53.5 days and 134 days, respectively. Four patients (22.2%) experienced immune‐related adverse events, and none of the patients reported grade 3 or higher irAEs. Hyperthermia was followed by an increase in the number of tumour immune‐activated cells.

**Conclusions:**

HIT can provide survival benefits in patients with GITs by activating antitumour immune function and shows good safety and feasibility.

This study pioneered the use of WIRA whole‐body infrared hyperthermia combined with ICI therapy to treat GIT and verified the feasibility and safety of HIT. The final results showed a DCR of 55.6%, with a median PFS of 53.5 days, median OS of 134 days, and an irAE incidence of 22.2%. Therefore, we believe that HIT can exert multiple synergistic sensitisation effects, thereby providing clinical benefits to patients with advanced GITs, increasing overall safety, and improving patients' QOL.

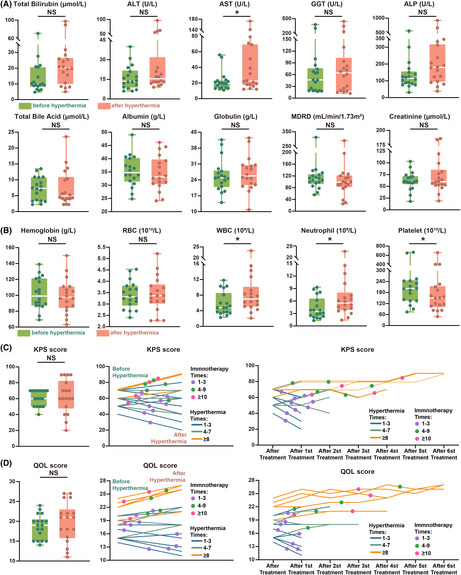

## 1 INTRODUCTION

Gastrointestinal tumours (GITs) are the most common and fatal cancers worldwide, accounting for 26% of all malignant tumours, and are associated with a mortality rate of 35%.^1^ Traditional surgery, chemotherapy and radiotherapy have poor overall efficacy on advanced GITs, with 5‐year survival rates of less than 5%.^2^ Immune checkpoint inhibitors (ICIs) can improve tumour immune suppression, restore the host's normal antitumour immune response, and eliminate cancer cells, thereby achieving therapeutic effects. However, in patients with GITs, due to differences in molecular pathology, only approximately 4% of patients with the high microsatellite instability (MSI‐H)/mismatch repair deficient (dMMR) phenotype can benefit from ICI treatment, which makes the application of ICIs particularly challenging.^3^ The KEYNOTE‐059 study showed that the objective response rates (ORR) of MSI‐H/dMMR and MSS/pMMR tumours to ICI treatment of gastric cancer were 57.1% and 9.0%, respectively.^4^ Similarly, the Checkmate‐032 study showed that MSI‐H tumours had an ORR nearly three times higher than that of MSS tumours (29% vs. 11%).^5^ Nevertheless, considering the small proportion of patients with the MSI‐H/dMMR phenotype, 96% of the patients exhibit an ‘immune desert’ tumour microenvironment (TME), thus resulting in few effective targets for ICI and deeply limiting its effectiveness. These findings highlight the need to continue exploring reliable solutions that can effectively improve the efficiency of ICI in patients with MSS/pMMR GITs.

Multimodal therapeutic modalities that aim to achieve high efficiency and low toxicity have emerged as an approach to improve the efficacy of single therapies. Hyperthermia is a rapidly developing new therapeutic approach in the field of oncotherapy, in which physical heating factors (infrared, microwave, radiofrequency and ultrasound, etc.) are used to raise the temperature of tumour tissue and suppress or kill tumour cells.^6^ Due to its low toxicity and high compatibility, it has become a crucial means of comprehensive cancer treatment. The underlying logic of clinical hyperthermia is the physio‐pathological differences in tumour tissues from normal tissues.^7^ Due to the deficiency of heat sensing and dissipating, the tumour lesions can generate a temperature more than 3°C higher than the normal tissues under hyperthermia.^8^ In turn, a complex cascade of thermal damage and multifactorial sensitization effects occurs, resulting in the following three modes of anti‐tumour rationale. Firstly, at the intracellular level, hyperthermia promotes the apoptosis of tumour cells.^9^ Due to the deviation from the optimal temperature, the efficiency of many organelles and sub‐organelles is weakened.^10^ Inhibition of certain transmembrane proteins disrupts ion transport, causing intracellular accumulation of Ca^2+^, which leads to endoplasmic reticulum stress and caspase‐dependent apoptosis.^11^ Secondly, from an immunological perspective, hyperthermia significantly enhances anti‐tumour immune responses, encompassing both specific and non‐specific immunity.^12^ Research has demonstrated that heat shock protein (HSP), tumour‐specific antigen (TSA), chemoattractant cytokine ligand (CCL) and CXC chemokine ligand (CXCL) stimulate the maturation, differentiation, migration and cytotoxicity of NK cells and macrophages.^13^, ^14^, ^15^ Hyperthermia impedes lymphatic homing, enhances tumour chemotaxis and augments cytotoxic T lymphocyte (CTL) mediated tumour cell killing, thereby bolstering specific anti‐tumour immunoreactivity.^16^ Furthermore, it suppresses Treg cell differentiation and fosters memory T cell generation through increased IL‐6 secretion by tumour infiltrating lymphocyte (TIL).^17^ Thirdly, hyperthermia relieves the drug‐resistant tumour microenvironment by increasing drug targets and their aggregation through upregulation of receptor expression, which further enhances the advantages of anticancer pharmacokinetics.^18^ The above mechanisms provide a compelling theoretical basis for integrating hyperthermia into multimodal anti‐tumour therapy, especially for gastrointestinal cancer patients with MSS/pMMR phenotype. We performed bioinformatics analysis using the TCGA database (https://portal.gdc.cancer.gov/) before conducting this clinical trial and confirmed that the release of pro‐inflammatory molecules after heat shock in GITs could significantly improve immune infiltration, with significant increments in the numbers of TILs, NK cells and DCs and a reduction in the number of inhibitory immune cells (Figure S1A–C). In the Kaplan–Meier analysis of overall survival (OS) for patients receiving PD‐1 antibody treatment with different levels of heat stress‐related molecule expression, the survival curve demonstrated that hyperthermia can enhance the efficacy of ICI by improving tumour immune suppression and providing practical survival benefits to patients (Figure S1D–S).^19^, ^20^, ^21^, ^22^ Considering the evidence available for benefits of HIT for patients with advanced cancer, we believe that the use of HIT for GITs has substantial exploratory potential for real‐world application.

The water‐filtered infrared A radiation (WIRA) hyperthermia system was developed by the German biophysicist G Hoffmann and has become one of the most advanced whole‐body hyperthermia devices in the world. The WIRA system blocks infrared B and C irradiation through a water‐filtration device, causing only a mild increase in skin temperature when used for deep tissue heating and thereby considerably increasing patient tolerance and the feasibility of hyperthermia for deep tumours (Figure S2).^23^ The HECKEL 3000MT‐4 T whole‐body hyperthermia system (HECKEL Medizintechnik, Germany) used in this study uses the aforementioned WIRA mechanism. The transmitter generates a stable infrared heat source with a wavelength range of 0.5–1.4 μm, thus ensuring that the skin temperature does not significantly increase while achieving a rapid rise in core temperature.

Currently, to our knowledge, there are no prospective clinical trials of HIT reported in the literature. In the light of the theoretical basis outlined above, we performed HIT treatment using a combination of WIRA whole‐body hyperthermia and a PD‐1 monoclonal antibody for GITs and aimed to evaluate and validate the clinical application prospects of HIT from the perspectives of efficacy, safety and improvement in quality of life (QOL). This study presents new inspiration and ideas for the treatment of advanced GITs and provides reliable evidence for the real‐world clinical application of HIT.

## 2 METHODS

### 2.1 Study design and participants

**FIGURE 1:**
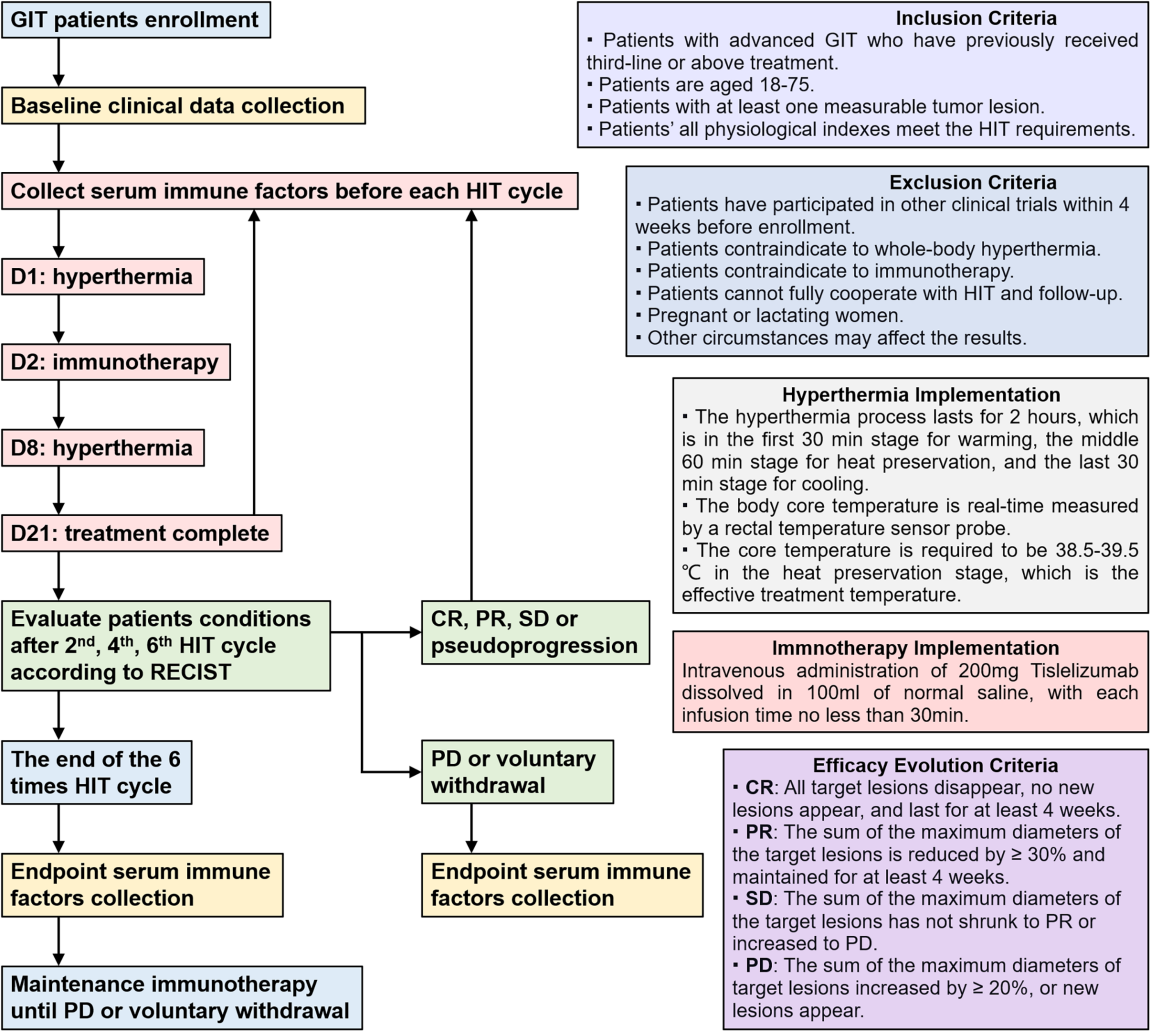
Trial profile. Flow sheet of HIT, inclusion and exclusion criteria, immunotherapy and hyperthermia protocols, and efficacy evaluation criteria.

This study was an investigator‐initiated, open‐label, single‐centre and single‐arm, prospective phase 2 clinical trial. The study population was selected from patients with GITs who visited the Oncology Department of the Affiliated Zhejiang Hospital, Zhejiang University School of Medicine, between 1 June 2020 and 31 May 2022. The inclusion and exclusion criteria are shown in Figure 1.

The specific treatment process is shown in the trial flow diagram (Figure 1). The patients underwent WIRA whole‐body hyperthermia on days 1 and 8 of each HIT cycle. On day 2 (24 h after hyperthermia on day 1), 200 mg of tislelizumab (BeiGene, China) prepared with 100 mL of normal saline was intravenously administered for less than 30 min. After six HIT cycles, tislelizumab was administered intravenously every 21 days until dropout. For quality control of hyperthermia, the core temperature was set to 38.5–39.5°C and measured using a rectal temperature‐sensing probe. Hyperthermia was considered to have been achieved when this temperature range was reached and maintained for 60 min. Each hyperthermia session lasted for 2 h, including a 30‐min heating stage, a 60‐min insulation stage, and a 30‐min cooling stage. Clinical data were collected every two HIT treatment cycles and evaluated using the RECIST version 1.1 standard (Table S1).

### 2.2 Outcomes

The primary outcome was the disease control rate (DCR), while the secondary outcomes were progression‐free survival (PFS), OS, safety and improvement in QOL. The safety analysis mainly focused on hyperthermia‐related adverse events and irAEs. The QOL assessments were primarily based on the Karnofsky Performance Status (KPS) (Table S2) and QOL (Table S3) scores.

### 2.3 Statistical analyses

Statistical analysis was performed using SPSS 25.0, with *p* < 0.05 indicating statistically significant differences. An Excel database was established, and the original data were entered. Single‐factor analysis of variance was used to compare groups with a normal distribution, while the rank‐sum test was used to compare non‐normally distributed measurement data between groups. In the paired design, if the difference was normally distributed, a paired *t*‐test was used; if the difference was skewed, the paired signed‐rank‐sum test was used. The 95% confidence intervals (CIs) were calculated based on a binomial distribution using the Clopper‐Pearson method. PFS and OS were analysed using the Kaplan–Meier method. The graphics (box plot, bar plot, survival curve, Sankey diagram, heat map) were plotted using GraphPad Prism 9, RAWGraphs 2.0 and Adobe Illustrator.

### 2.4 Role of the funding source

The funding agency of the study had no role in the study design, data collection, data analysis, data interpretation, or writing the manuscript.

## 3 RESULTS

Between 1 June 2020 and 31 May 2022, 18 patients (12 males; six females) were enrolled in the study (Table 1). As of 19 May 2023, 17 of the 18 patients had completed follow‐up, and one patient was still undergoing follow‐up.

**TABLE 1:** Baseline patient and disease characteristics.

Baseline characteristics	Total (*n* = 18)
Age (years)	57 (30–73)
Sex	Male (12, 66.7%)
	Female (6, 33.3%)
Race	Asian (18, 100%)
Disease	Gastric cancer (6, 33.3%)
	Colon cancer (7, 38.9%)
	Rectal cancer (3, 16.7%)
	Appendiceal cancer (2, 11.1%)
ECOG score	1 (6, 33.3%)
	2 (10, 55.6%)
	3 (2, 11.1%)
Lymphatic metastasis	14 (77.8%)
Liver metastasis	10 (55.6%)
Peritoneal metastasis	14 (77.8%)
Previous surgery	16 (88.9%)
Previous chemotherapy	18 (100%)
Previous targeted therapy	12 (66.7%)
Number of previous therapies	
3	4 (22.2%)
4	4 (22.2%)
5	5 (27.8%)
> = 6	5 (27.8%)

As shown in Figure S3A–D, the overall temperature curve satisfied the quality control requirements for hyperthermia, reaching the treatment temperature within approximately 30 min, and the core temperature fluctuated steadily within the treatment temperature range subsequently. All 18 patients were able to tolerate the 60‐min heat insulation stage, and changes in respiration, blood pressure, and heart rate were closely correlated with the core temperature (Figure S3E–G). As shown in Figure S3H,I, the oxygen flow increased with an increase in the core temperature, leading to an oxygen saturation above 98%. Each patient tolerated the temperature changes during the heating and cooling stages well. The overall controllability of temperature during the treatment process was high, and the heat distribution in the chamber was uniform, indicating the feasibility of hyperthermia.

**FIGURE 2:**
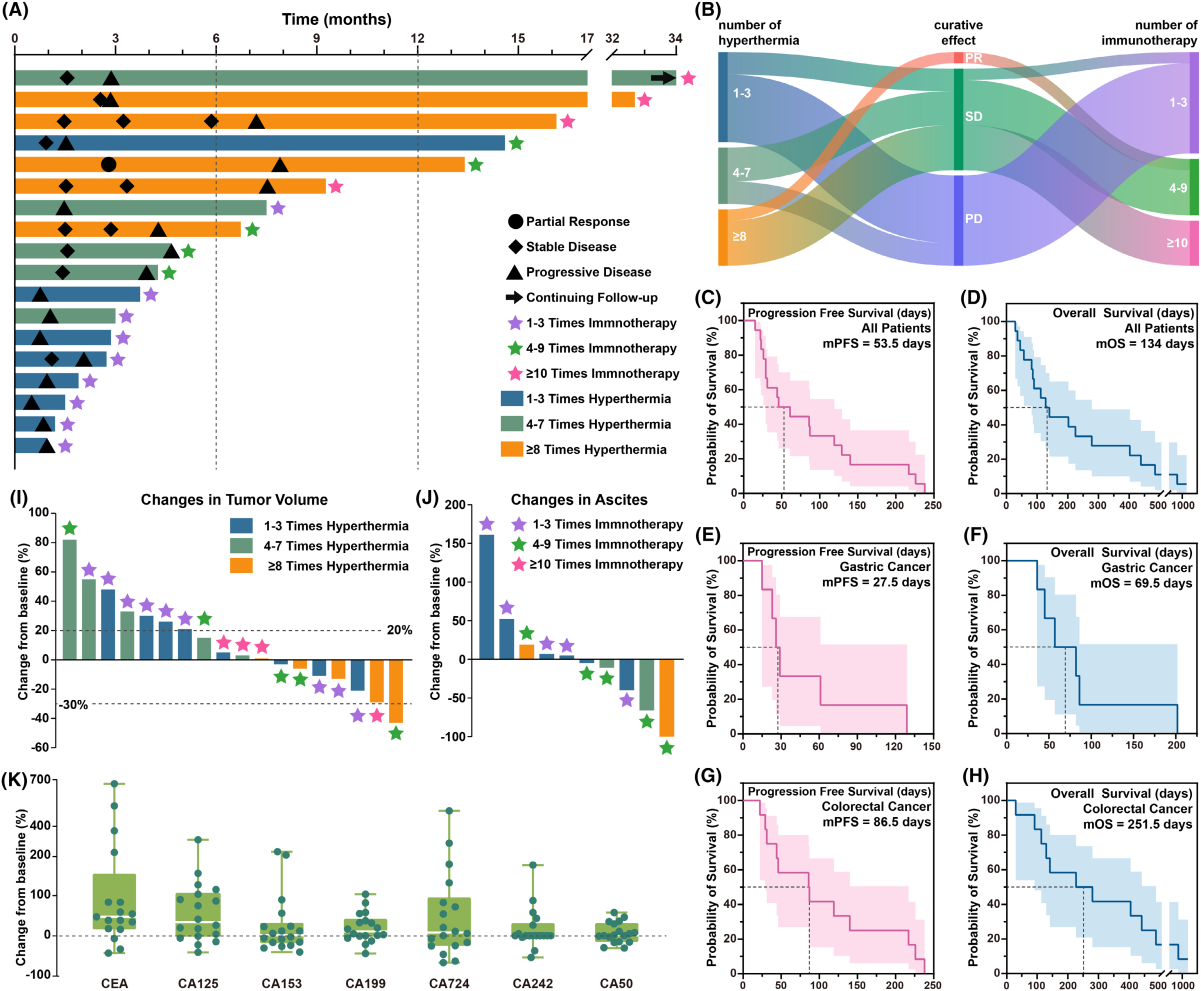
Curative effect of hyperthermia combined with immune checkpoint inhibitors. (A) Swimmer plot. (B) The correspondence between different hyperthermia times, immunotherapy times, and curative effect. (C) The progression free survival of all patients. (D) The overall survival of all patients. (E) The progression free survival of gastric cancer patients. (F) The overall survival of gastric cancer patients. (G) The progression free survival of colorectal cancer patients. (H) The overall survival of colorectal cancer patients. (I) The comparison of tumour volume before and after treatment. (J) The comparison of ascites volume before and after treatment in the 10 patients with ascites. (K) The changes of before and after HIT of CEA, CA125, CA153, CA199, CA724, CA242, and CA50.

As of 19 May 2023, 17 patients had died, including 14 deaths due to disease progression and three deaths due to disease progression combined with intestinal infections, acute renal failure, or stroke (Figure 2A). There were 8 patients completed 1–3 times of hyperthermia, 5 completed 4–7 times and 5 completed ≥8 times. Nine patients completed 1**–**3 times of immunotherapy, 5 completed 4–7 times, and 4 completed ≥8 times. DCR, the primary endpoint, was 55.6% (95% CI, 30.8% to 78.5%), with the subgroup analysis showing a DCR of 33.3% (95% CI, 4.3% to 77.7%) for gastric cancer and 60.0% (95% CI, 34.9% to 90.1%) for colorectal cancer (Table 2). The correspondence relationship between treatment times and the curative effect is shown in Figure 2B. Six patients (33.3%; 95% CI, 13.3%–59.0%) achieved a 3‐month PFS, while three (16.7%; 95% CI, 3.6%–41.4%) achieved a 6‐month PFS, and the median PFS was 53.5 days (95% CI, 49.8–124.5) (Figure 2C). Twelve patients (66.7%; 95% CI, 41.0%–86.7%) achieved a 3‐month OS; eight (44.4%; 95% CI, 21.5%–69.2%) achieved a 6‐month OS; and five (27.8%; 95% CI, 9.7%–53.5%) achieved a 1‐year OS, with a median OS of 134 days (95% CI, 119.1–417.7) (Figure 2D). Subgroup analysis showed that the median PFS of patients with gastric cancer was 27.5 days (95% CI, 1.9–92.4), and the median OS was 69.5 days (95% CI, 20.9–148.5) (Figure 2E,F) The median PFS of patients with colorectal cancer was 86.5 days (95% CI, 55.6–158.6), and the median OS was 251.5 days (95% CI, 149.5–571.0) (Figure 2G,H). The waterfall plots to compare the tumour and ascites volumes before and after HIT are shown in Figure 2I,J, and the differences in tumour markers as in Figure 2K.

**TABLE 2:** Data of the primary endpoint.

Efficacy Parameter	Data
All Patients (*n* = 18)	
Compete response	0
Partial response	1
Stable disease	9
Progressive disease	8
Disease control rate	55.6% (95% CI, 30.8% to 78.5%)
Gastic cancer (*n* = 6)	
Compete response	0
Partial response	0
Stable disease	2
Progressive disease	4
Disease control rate	33.3% (95% CI, 4.3%–77.7%)
Colorectal cancer (*n* = 10)	
Compete response	0
Partial response	1
Stable disease	5
Progressive disease	4
Disease control rate	60.0% (95% CI, 34.9%–90.1%)

**FIGURE 3:**
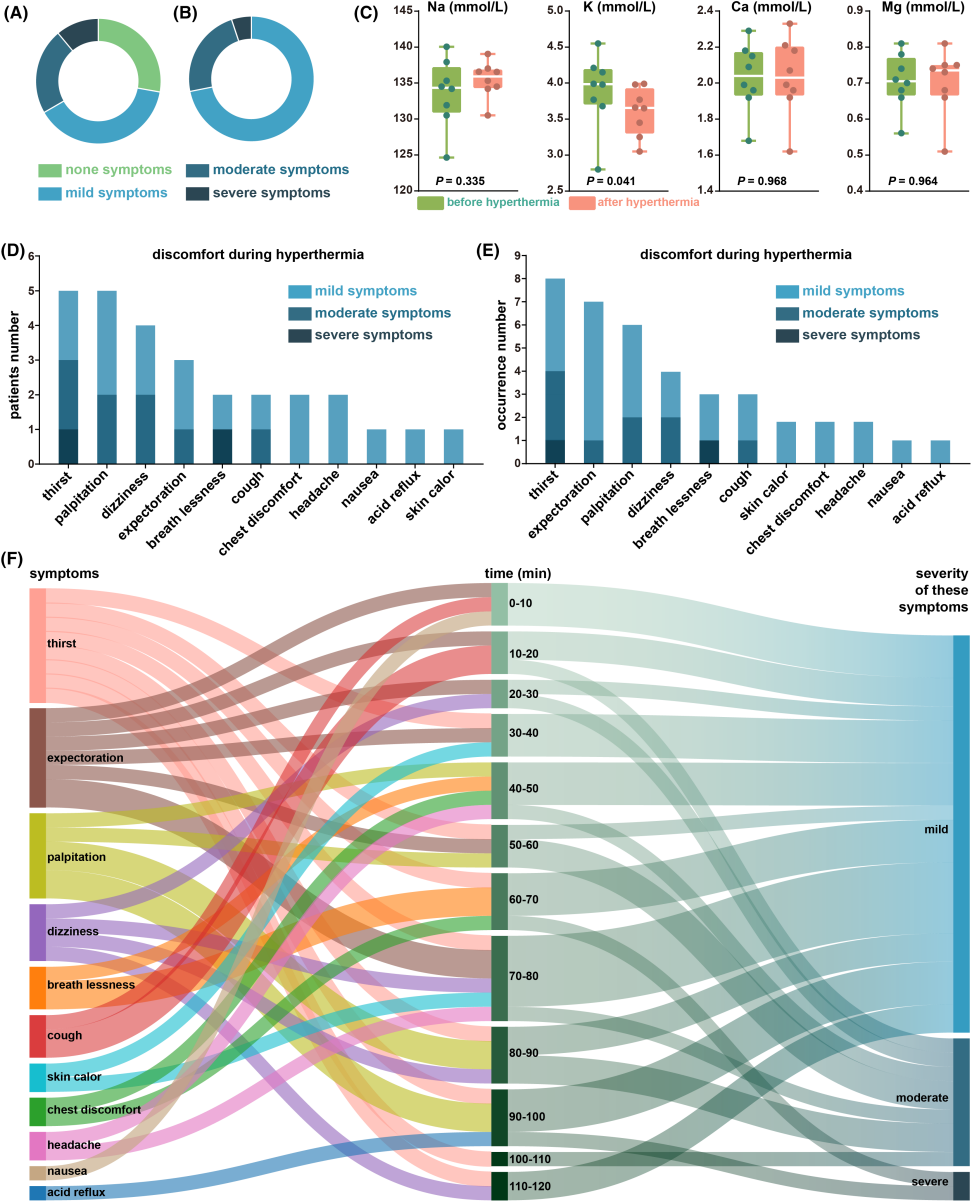
The incidence of hyperthermia related adverse events of the 18 patients. (A) Patients numbers of hyperthermia related adverse events at all grades. (B) Patients numbers of hyperthermia related adverse events at all grades. (C) Electrolytes changes in patients with thirst. (D) Patients numbers of hyperthermia related adverse events of various types and grades. (E) Patients numbers of hyperthermia related adverse events of various types and grades. (F) Correspondence between types, grades and occurrence times of hyperthermia related adverse events.

**FIGURE 4:**
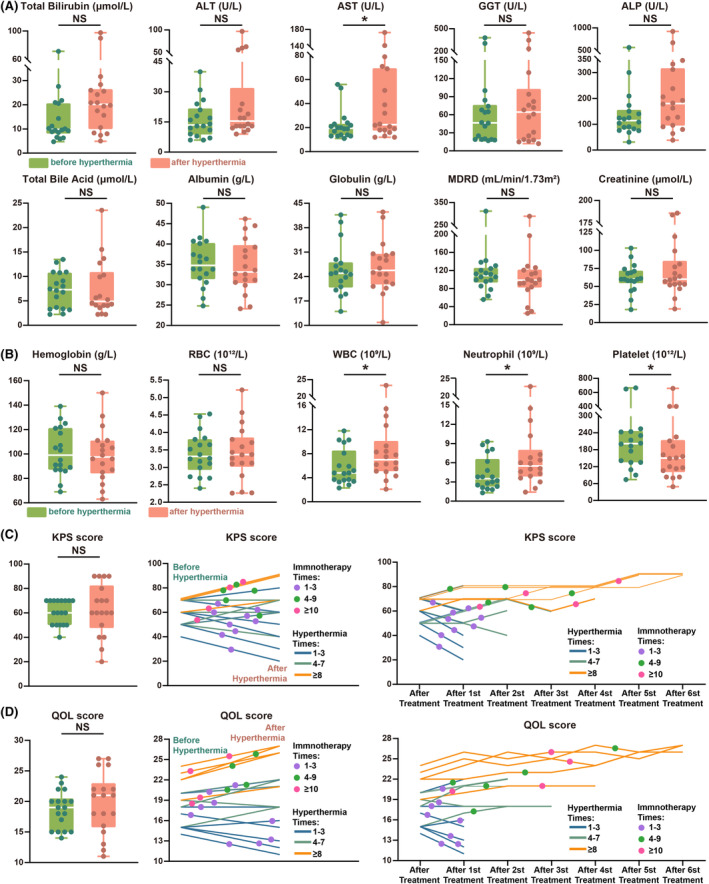
The safety and improvement in quality of life effect of HIT. (A) Liver function and renal function test. (B) Blood routine test. (C, D) The changes of KPS score and QOL score before and after HIT. *p* < 0.05.

**FIGURE 5:**
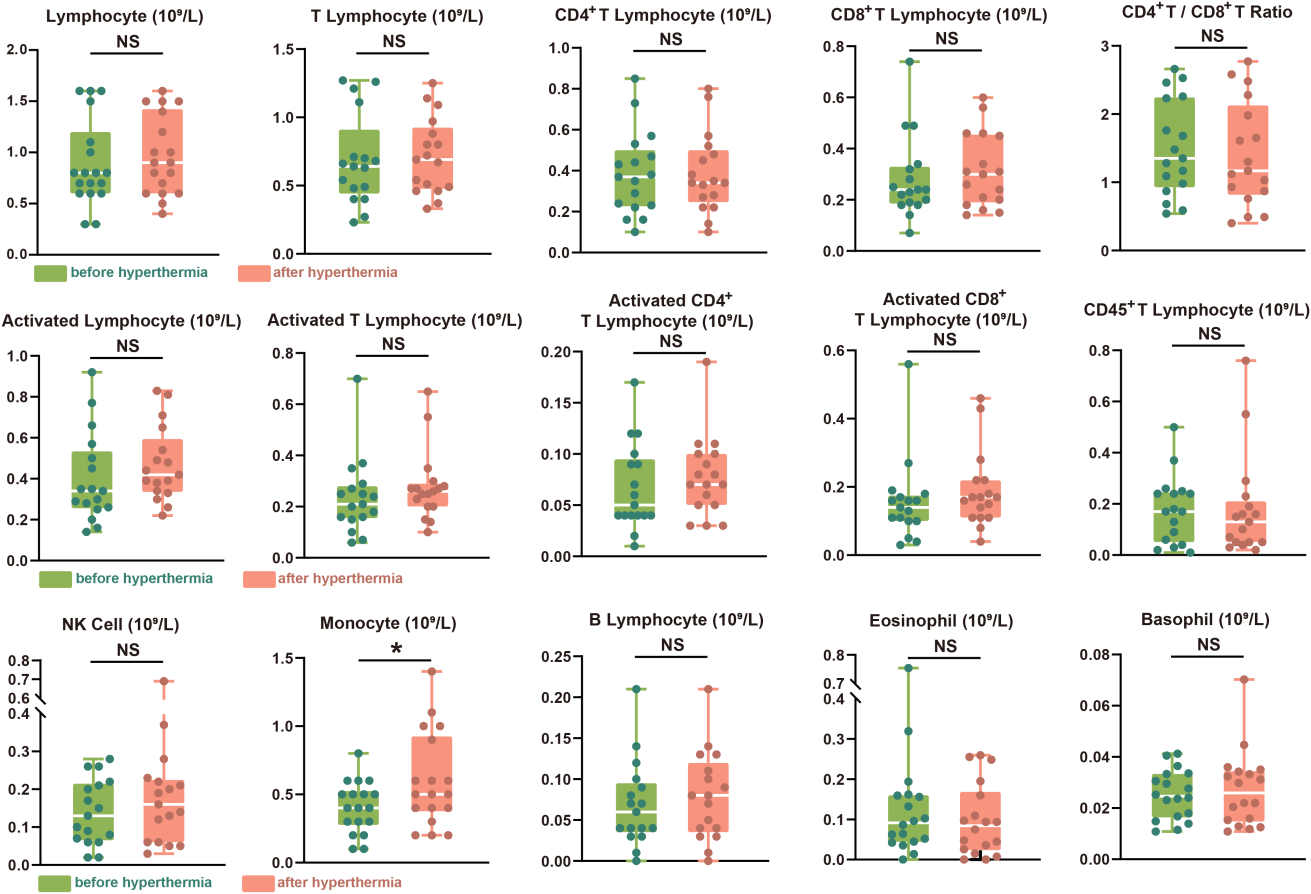
Analysis of immune cell subtypes. The changes of various immune cells before and after HIT. *p* < 0.05.

Close monitoring of the adverse reactions in the patients during the hyperthermia sessions revealed that 13 patients (72.2%) experienced at least one episode of discomfort across all hyperthermia treatment sessions, as shown in Figure 3A. In the trial design phase, we preliminarily defined the meaning and severity of the hyperthermia safety indicators, and in the subsequent trial conduct phase, we concretized the 11 observed adverse events, as shown in Table S4. As shown in Figure 3B, out of the 93 hyperthermia sessions, 39 (41.9%) resulted in hyperthermia related discomfort symptoms. Thirst caused by excessive sweating was the most commonly reported adverse event (AE). In patients with excessive sweating, changes in the main electrolytes were observed before and after hyperthermia, as shown in Figure 3C. In addition to thirst, the common adverse events are palpitation, expectoration and dizziness, which are controllable (Figure 3D,E). As shown in Figure 3F, the period from 70 to 90 min during hyperthermia was the most common period for the occurrence of AEs. All symptoms of discomfort quickly relieved after hyperthermia, and no persistent AEs were observed. None of the patients terminated hyperthermia treatment or abandoned the trial because of these AEs. From the first session of immunotherapy to the follow‐up period of 6 months after the last session of immunotherapy or death, as shown in Table S5, among the patients, one stopped hyperthermia due to irAEs (fever), no patients died or discontinued tislelizumab due to irAEs. In addition to evaluating the AEs related to HIT, we also recorded changes in various important blood indicators before and after HIT, as shown in Figure 4A,B. Besides, the KPS and QOL scores before and after HIT were investigated (Figure 4C,D), and the results showed that both the KPS and QOL scores improved; however, the differences were not statistically significant. Finally, we analysed the blood immune cell subtypes of participants before and after HIT to explore the mechanisms underlying the synergistic effects of HIT (Figure 5). We found increased levels of a variety of tumour immunoactivated cells after HIT.

## 4 DISCUSSION

This study explored a method to improve the efficacy of immunotherapy in patients with GITs having the MSS/pMMR phenotype. Based on the synergistic mechanisms outlined in a large number of studies on HIT, we conducted this real‐world prospective study on the use of HIT for GITs with the MSS/pMMR phenotype and obtained encouraging preliminary results.

Prospective clinical trials targeting GITs tend to include patients with an ECOG score of 0–1, while patients with an ECOG score of ≥2 are rarely able to participate in prospective clinical trials related to immunotherapy.^24^, ^25^ Differently, 12 patients (67%) with ECOG score ≥2 are being employed in our trial to explore the effectiveness and security of HIT. All patients had metastases with TNM staging IV and received at least three lines of treatments. Specifically, 16 cases (88.9%), 18 cases (100%) and 12 cases (66.7%) had previously received surgery, chemotherapy and targeted therapy, respectively. These patients had developed resistance to all these treatments, which greatly limited their therapeutic options. 8 patients provided complete immunohistochemical profiles of mismatch repair gene expression, all exhibiting MSS/pMMR phenotypes. 5 patients provided HER‐2 expression information, with three being (−) and two being (++). 2 patients provided PD‐L1 CPS scores, both <1%. These demographic characteristics suggest greater treatment challenges and poorer prognosis.

HIT still provided some survival benefits in these patients, despite these 18 patients generally had poor conditions in comparison with the patient populations in other large‐scale clinical studies. The ATTRACTION‐2 study was a double‐blind, randomised controlled, phase III clinical trial that enrolled an East Asian population, in which 163 patients with advanced gastric cancer received placebo treatment.^26^ The results showed that the DCR of the placebo group in ATTRACTION‐2 was lower than our HIT data (25.0% vs. 33.3%). Similarly, the KEYNOTE‐059 study administered pembrolizumab monotherapy to 259 patients with gastric cancer who received second‐line or higher treatment.^4^ Their results showed a DCR of 27%, which was lower than that observed in our study (55.6%). These findings indirectly confirmed the synergistic effects of HIT. However, the OS and PFS rates in the KEYNOTE‐059 study were higher than those in our study, which we believe was related to the differences in the inclusion criteria used in the two studies. The KEYNOTE‐059 trial included only patients with an ECOG score of 0–1. In contrast, all patients enrolled in our trial had stage IV disease, showed an ECOG score of 1–3, and had previously received at least three lines of treatment. Thus, the overall severe illness and poor general condition of our patients are important reasons for the slightly lower OS in our study than in KEYNOTE‐059. Kuang et al. conducted a single‐arm clinical trial of azacitidine combined with pembrolizumab for treating metastatic colorectal cancer.^27^ The results showed that the median PFS was 1.9 months and the median OS was 6.3 months, both lower than the survival period in our study. In addition to chemotherapy, immunostimulants have also been evaluated as synergistic adjuncts to ICI, such as the combination of PexaVec and ICI for the treatment of advanced colorectal cancer.^28^ The survival data of that trial showed a PFS and OS of 2.3 and 7.5 months, respectively, in the PexaVec/Durva group, while the PFS and OS in the PexaVec/Durva/Treme group were 2.1 and 5.2 months, respectively; both of these values were lower than the PFS and OS in our study. We believe that the specific mechanisms underlying HIT synergism are closely related to host immune stimulation caused by whole‐body hyperthermia.

Hyperthermia is a mild therapy with relatively low toxicity; however, monitoring adverse reactions remains a focal point of clinical trials. The risk associated with hyperthermia is closely tied to its operational temperature. Studies indicate that ablating solid tumours can lead to various complications, including bleeding, infection, local persistent pain, and impairment of organ function.^29^, ^30^ Furthermore, localised mild hyperthermia such as microwave hyperthermia, hyperthermic intraperitoneal chemotherapy, and bladder perfusion chemotherapy have been reported to cause skin, mucosal, and soft tissue burns due to uneven distribution of body temperature.^31^, ^32^, ^33^ Continuous and repeated heating could elevate the risk of catarrh, particularly when combined with chemotherapy.^34^ Nevertheless, adverse events related to hyperthermia remain mild and controllable compared to conventional treatments. Unlike medications, hyperthermia does not undergo molecular metabolism in the liver or kidneys but by heat conduction and dissipation. Its adverse reactions typically stem from high‐temperature damage to normal tissues, underscoring the importance of precise control over the scope and temperature in hyperthermia applications. While the WIRA technique employed in this trial provided a certain level of safety assurance, given the extensive treatment range and prolonged treatment duration of whole body hyperthermia, we incorporated various indicators (vital signs, multi‐site temperature, subjective discomfort) into the experimental design phase to meticulously monitor the occurrence of hyperthermia related adverse events. Although the results suggest that the overall safety of hyperthermia is good, patients still experienced discomfort with the increase in the core temperature, especially during the 70–90 min period when the temperature reached its peak. For patients who have already experienced severe discomfort at a certain core temperature, preventive measures, such as appropriately lowering the temperature, increasing oxygen flow, and increasing water intake before hyperthermia, can be taken during the next hyperthermia session. irAEs were another important observational indicator in this study, in addition to efficacy, and are also known to be a difficult and key point in current studies on oncotherapy. The common irAE symptoms of PD‐1 inhibitors, which mainly include dermatitis, hypothyroidism, pneumonia, liver function damage, fever and colitis, are summarised in Figure S4.^35^, ^36^ Several types of symptoms that appeared in this study were common irAEs, but the difference was that the incidence of irAEs in 18 patients was 22.2%, which was significantly lower than the incidence of irAEs after anti‐PD‐1 therapy in a large‐sample meta‐analysis.^37^ Several studies, including KEYNOTE‐059 (77.4%), KEYNOTE‐061 (53%), and KEYNOTE‐062 (54.3%), have shown that the incidence of irAEs in single‐drug ICI therapy is >50%.^38^, ^39^, ^40^ We attributed the lower incidence of irAEs is the improvement in aggregation of ICI and immune cells tumour‐chemotaxis under hyperthermia. Kleef et al. validated this mechanism.^41^ In their retrospective analysis of 131 patients with solid tumours treated with HIT, the overall incidence of irAEs was 51.9%. Laboratory blood tests showed a statistical difference in AST elevation and platelet reduction, which may indicate a potential safety hazard of HIT. While the reasons for these data, we reckon that is multi‐faceted, the most important is the poor condition in the baseline. All of the 18 patients had received chemotherapy, and 10 (55.6%) with liver metastases, which resulted in severe irreversible damage to the livers and bone marrow. However, there was no statistical difference in the other indicators. Therefore, we believe that HIT will not aggravate or directly cause more dangerous liver and bone marrow injury.

To further analyse the potential mechanisms underlying the therapeutic advantages of HIT and to validate the synergistic sensitisation mechanism, we selected multiple immune indicators closely related to host tumour immune function for analysis. We found that the cellular immune mechanism associated with good efficacy was related to the proliferation and activation of immunostimulatory cells, thereby providing real‐world validation of the synergistic mechanism of HIT. In terms of specific immunity, patients with MSS/pMMR GITs usually show immune inhibition, but hyperthermia can stimulate the activation and maturation of APCs and thereby influence the differentiation direction and phenotypic changes of different subtypes of T cells.^43^ In this study, the number of monocytes increased significantly after hyperthermia treatment, which further increased the possibility of tumour infiltration by more DCs and APCs. With the release of TSA after hyperthermia, APCs induce greater CTL activation. We observed a slight increase in the numbers of activated lymphocytes, activated T lymphocytes, activated CD4^+^ T lymphocytes, and activated CD8^+^ T lymphocytes after hyperthermia. Due to the limited enrollment and data, most results on immune cell subtypes were not statistically significant. However, these mild elevations still tend to a positive inducing and effecting in immune stimulation to some extent. This heat stress‐induced immune activation phenomenon was reflected in non‐specific immunisation, and the data showed an increase in NK cells and neutrophils. In addition, due to the use of whole‐body hyperthermia as an immune stimulation pretreatment in this study, we also observed an abscopal effect caused by the ‘tumour vaccine’, which showed excellent therapeutic effects on metastatic lesions. One patient enrolled in our trial had peritoneal metastasis accompanied by a large amount of malignant ascitic fluid. However, after HIT treatment, the ascites was significantly reduced and maintained, as shown in Figure S5. This phenomenon has also been reported in previous studies on HIT.^42^, ^43^ Kleef et al. reported a case of stage IV triple‐negative breast cancer with lung metastasis in which the patient received nivolumab + ipilimumab and whole‐body hyperthermia therapy and finally, showed complete response of the lung metastasis with a survival period of 27 months.^44^ However, single‐arm studies lack control and cannot avoid non‐random selection bias. The sample size of our trial was limited, and the detailed indicators (correlative factors, prediction index, subgroup analysis) about survival and safety in HIT would require in‐depth research in future large‐scale studies.

In summary, this study pioneered the use of WIRA whole‐body infrared hyperthermia combined with ICI therapy to treat GIT and verified the feasibility and safety of HIT. The final results showed a DCR of 55.6%, with a median PFS of 53.5 days, median OS of 134 days, and an irAE incidence of 22.2%. Therefore, we believe that HIT can exert multiple synergistic sensitisation effects, thereby providing clinical benefits to patients with advanced GITs, increasing overall safety, and improving patients' QOL. This therapy is effective and feasible and warrants further in‐depth research and application on a larger scale and in larger patient populations in the future.

## AUTHOR CONTRIBUTIONS

**Pengyuan Liu:** Data curation (lead); formal analysis (lead); funding acquisition (equal); investigation (lead); software (lead); visualization (lead); writing – original draft (lead). **Jing Wu:** Data curation (equal); investigation (equal). **Liting Chen:** Funding acquisition (equal); investigation (equal). **Zhenhai Wu:** Data curation (equal); investigation (equal). **Yufei Wu:** Methodology (equal); software (equal). **Ganlu Zhang:** Data curation (equal); investigation (equal). **Bingqi Yu:** Data curation (equal); investigation (equal). **Beibei Zhang:** Data curation (equal); investigation (equal). **Nan Wei:** Data curation (equal); investigation (equal). **Jinan Shi:** Data curation (equal); investigation (equal). **Chuanfeng Zhang:** Data curation (equal); investigation (equal). **Lan Lei:** Data curation (equal); investigation (equal). **Shuhuan Yu:** Data curation (equal); investigation (equal). **Jianjun Lai:** Data curation (equal); investigation (equal); validation (equal). **Zhen Guo:** Data curation (equal); investigation (equal). **Yuli Zheng:** Data curation (equal); investigation (equal); project administration (equal). **Zhao Jing:** Formal analysis (equal); methodology (equal); project administration (equal). **Hao Jiang:** Data curation (equal); project administration (equal); validation (equal). **Tingxiang Wang:** Conceptualization (equal); data curation (equal); project administration (equal). **Jueyi Zhou:** Methodology (equal); software (equal). **Yajun Wu:** Methodology (equal); software (equal). **Chuan Sun:** Conceptualization (equal); software (equal); validation (equal). **Jie Shen:** Conceptualization (equal); project administration (equal); resources (equal). **Jian Zhang:** Conceptualization (equal); project administration (equal); resources (equal). **Zhibing Wu:** Conceptualization (lead); data curation (equal); funding acquisition (lead); methodology (lead); project administration (lead); resources (lead); supervision (lead); writing – review and editing (lead).

## CONFLICT OF INTEREST STATEMENT

The authors declare no potential conflicts of interest.

## ETHICAL APPROVAL AND CONSENT TO PARTICIPATE

This trial was approved by the Medical Ethics Committee of Zhejiang Hospital (AF/SC‐06/04.2). Informed consent was obtained from all individual participants included in the study. This trial has been registered at clinicaltrials (NCT06022692).

## Supporting information


Figure S1.

Figure S2.

Figure S3.

Figure S4.

Figure S5.

Table S1.

Table S2.

Table S3.

Table S4.

Table S5.

## Data Availability

All data generated or analysed during this study are included in this published article and its supplemental materials. Source data for the figures in this study are available from the corresponding author upon reasonable request.
