# Supplementary figures and images for: Water‐filtered infrared A radiation hyperthermia combined with immunotherapy for advanced gastrointestinal tumours
Source: Cancer Med. 2024 Jul 24;13(14):e70024. doi: 10.1002/cam4.70024 (PMC11269209; doi:10.1002/cam4.70024)

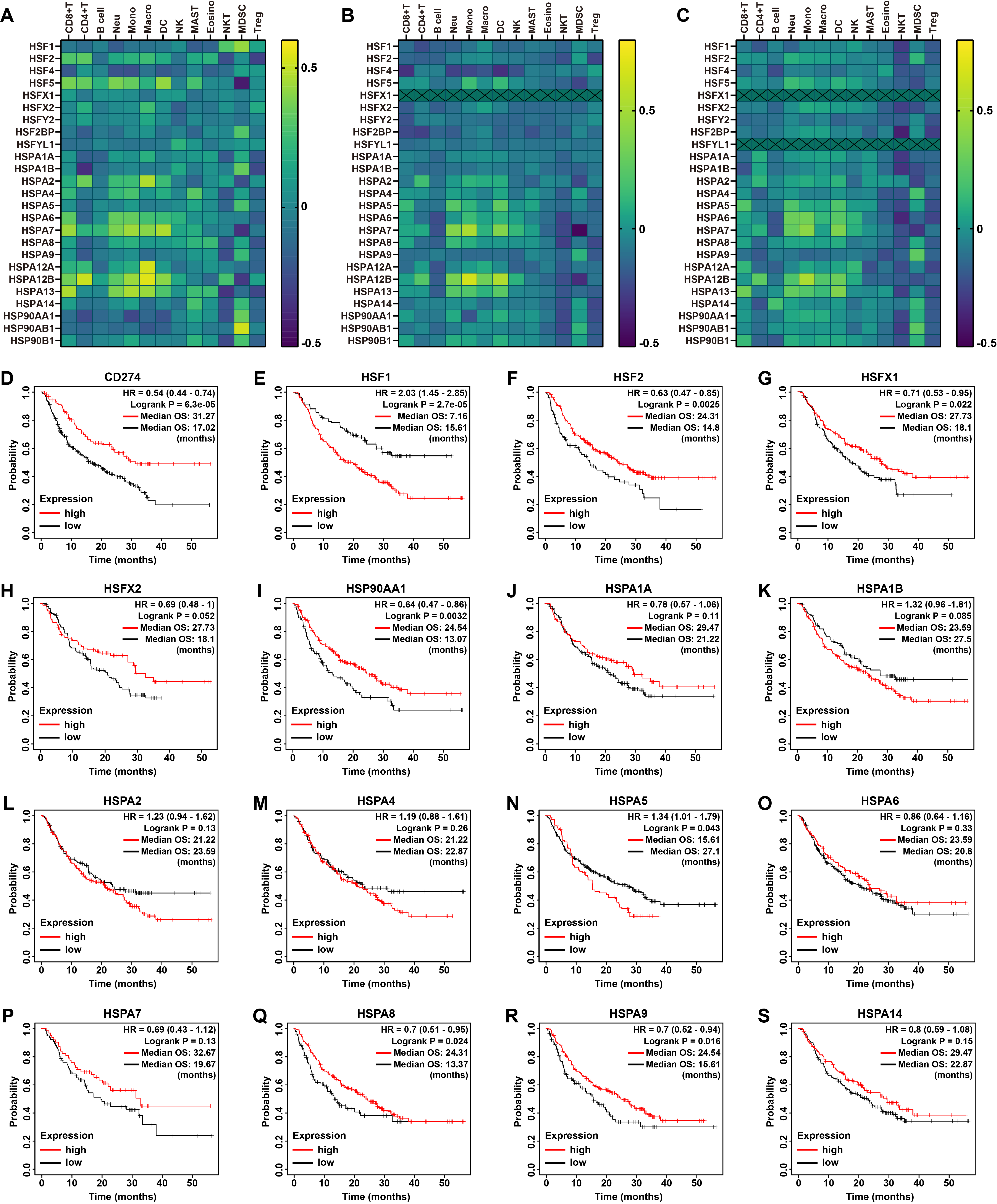

Supplement: Supplementary file 1 — Figure S1. [file CAM4-13-e70024-s001.png]

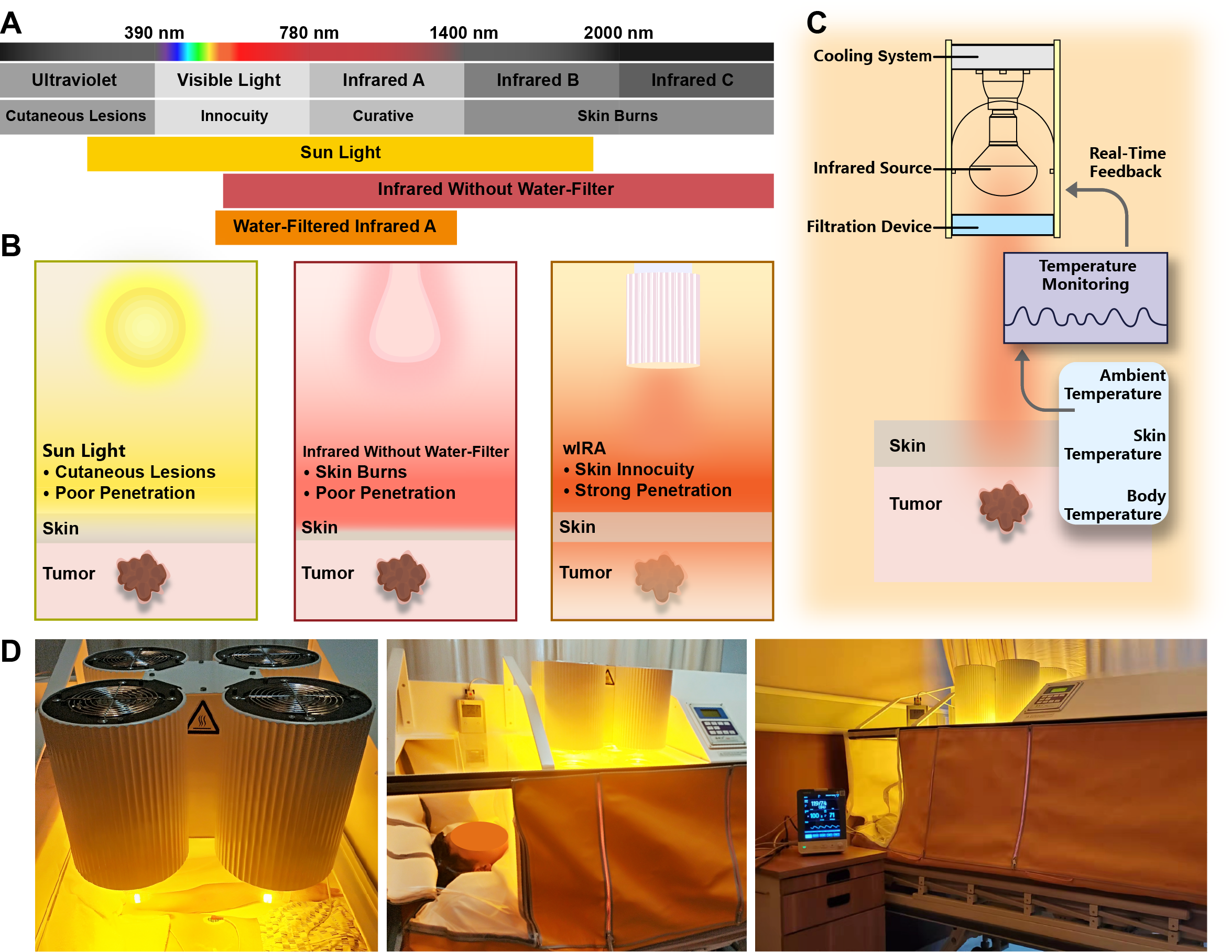

Supplement: Supplementary file 2 — Figure S2. [file CAM4-13-e70024-s007.png]

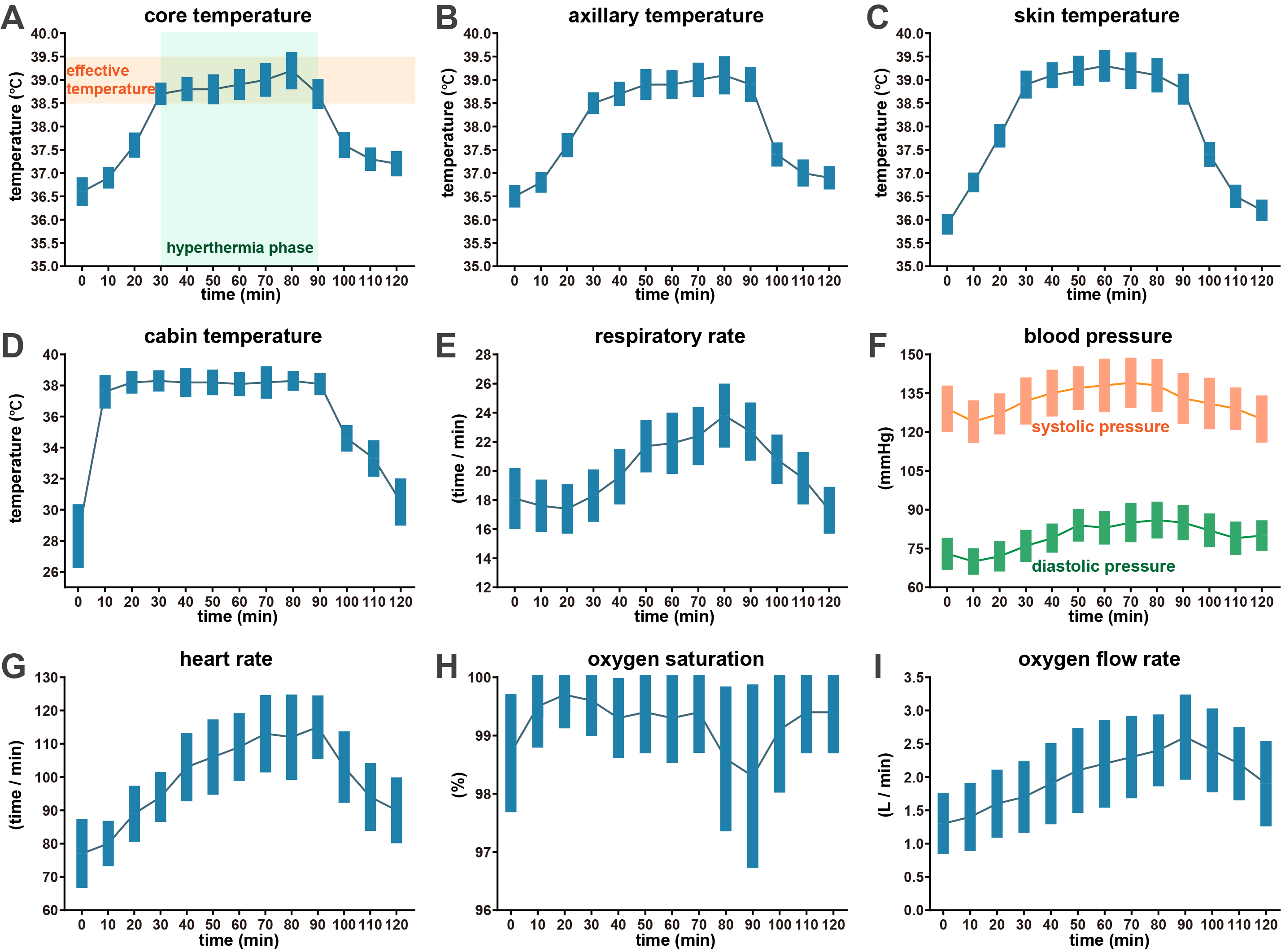

Supplement: Supplementary file 3 — Figure S3. [file CAM4-13-e70024-s005.png]

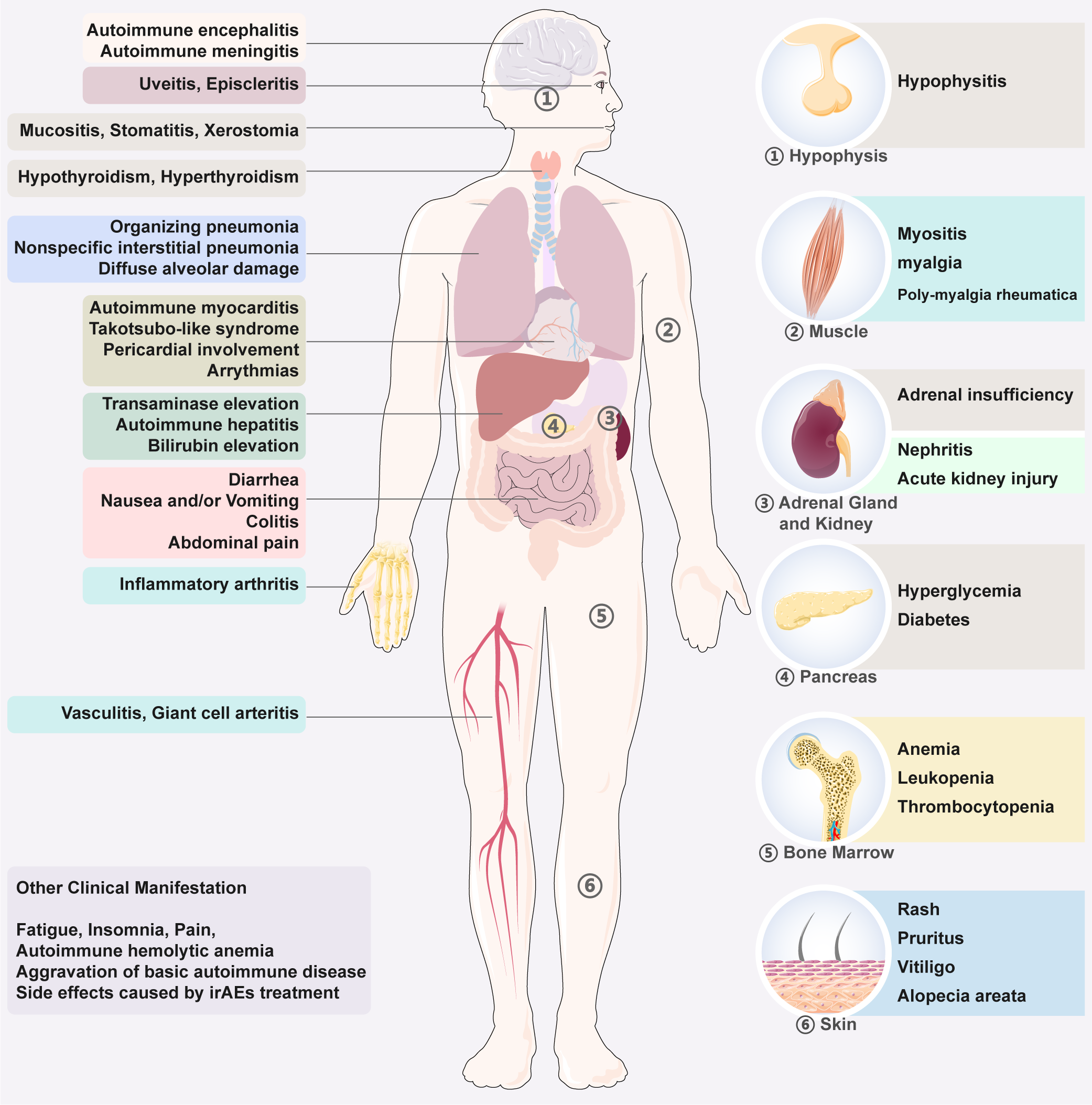

Supplement: Supplementary file 4 — Figure S4. [file CAM4-13-e70024-s004.tif]

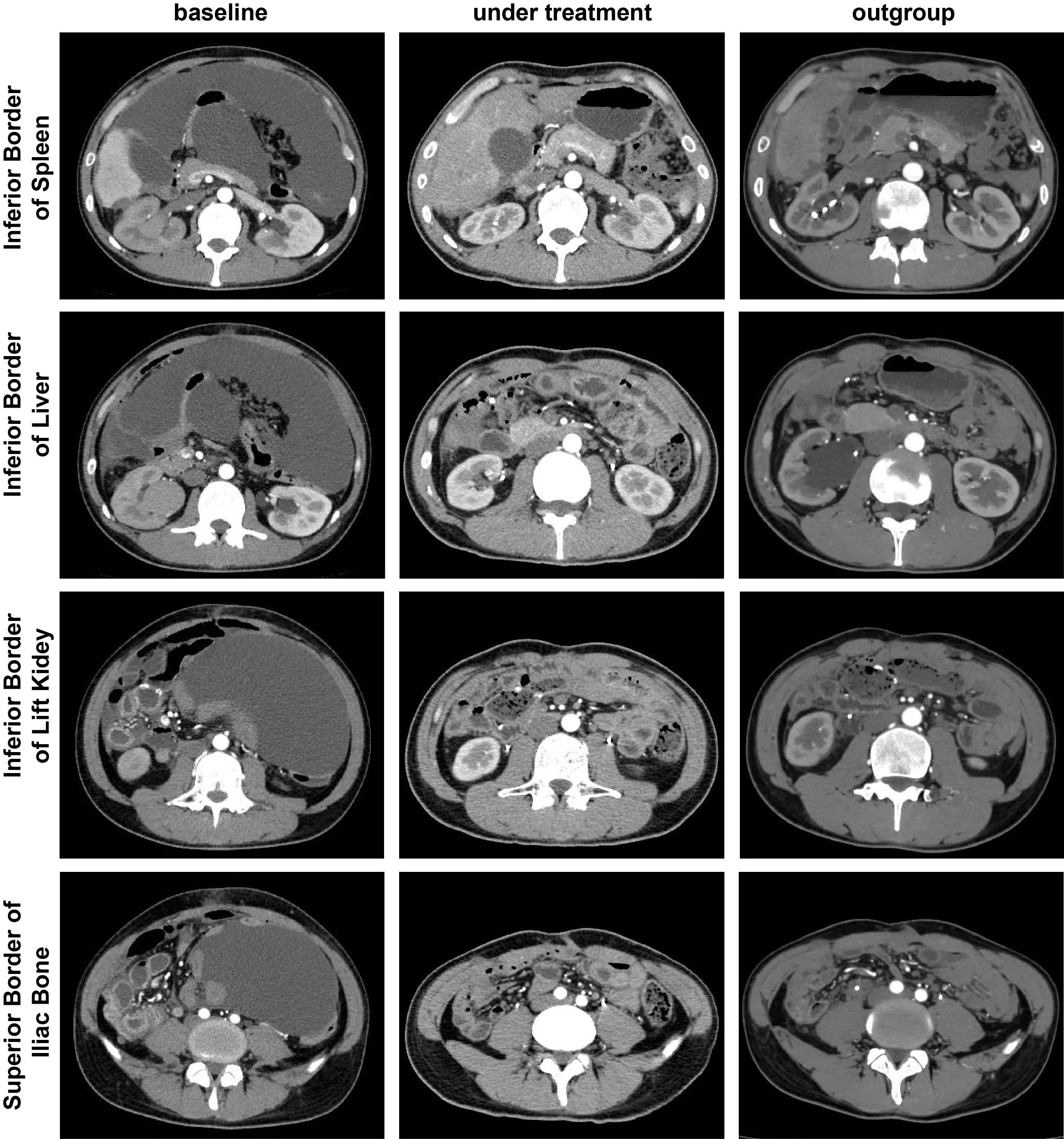

Supplement: Supplementary file 5 — Figure S5. [file CAM4-13-e70024-s003.png]
