# Supplementary material for: Water‐filtered infrared A radiation hyperthermia combined with immunotherapy for advanced gastrointestinal tumours
Source: Cancer Med. 2024 Jul 24;13(14):e70024. doi: 10.1002/cam4.70024 (PMC11269209; doi:10.1002/cam4.70024)
Supplement: Supplementary file 6 — Table S1. [file CAM4-13-e70024-s009.docx]

| **RECIST version 1.1 standard** | |
| --- | --- |
| **Compete Response** | All the tumors disappear and no new lesions occur for at least 4 weeks. |
| **Partial Response** | The sum of the maximum diameters of the tumors decreases by ≥ 30% and maintains for at least 4 weeks. |
| **Stable Disease** | The sum of the maximum diameters of the tumors decreases by less than PR or increases by less than PD. |
| **Progressive Disease** | The sum of the maximum diameters of the tumors increases by ≥ 20%, or new lesions occur. |

Supplementary Table 1
