# Supplementary material for: Water‐filtered infrared A radiation hyperthermia combined with immunotherapy for advanced gastrointestinal tumours
Source: Cancer Med. 2024 Jul 24;13(14):e70024. doi: 10.1002/cam4.70024 (PMC11269209; doi:10.1002/cam4.70024)
Supplement: Supplementary file 7 — Table S2. [file CAM4-13-e70024-s006.docx]

| **Karnofsky Performance Status** |  |
| --- | --- |
| **Description** | **Score** |
| Normal, no complaints or evidence of disease. | **100** |
| Able to perform normal activity; minor signs and symptoms of disease. | **90** |
| Able to perform normal activity with effort; some signs and symptoms of disease. | **80** |
| Cares for self, unable to perform normal activity or to do active work. | **70** |
| Requires occasional assistance but is able to care for most of own needs. | **60** |
| Requires considerable assistance and frequent medical care. | **50** |
| Requires special care and assistance; disabled. | **40** |
| Hospitalisation indicated, although death not imminent; severely disabled. | **30** |
| Hospitalisation necessary; active supportive treatment required, very sick. | **20** |
| Fatal processes progressing rapidly; moribund. | **10** |
| Dead | **0** |

Supplementary Table 2
