# Supplementary material for: Water‐filtered infrared A radiation hyperthermia combined with immunotherapy for advanced gastrointestinal tumours
Source: Cancer Med. 2024 Jul 24;13(14):e70024. doi: 10.1002/cam4.70024 (PMC11269209; doi:10.1002/cam4.70024)
Supplement: Supplementary file 8 — Table S3. [file CAM4-13-e70024-s002.docx]

| **Quality of Life Score** | | | | | |
| --- | --- | --- | --- | --- | --- |
| **Items** | **1** | **2** | **3** | **4** | **5** |
| **Appetite** | Inability to eat | Eating less than half of normal | Eating half the normal amount | Eating slightly less | Normal appetite |
| **Spirit** | Very poor | Poor | Sometimes poor, sometimes good | Slightly good | Good |
| **Sleep** | Insomnia | Difficulty falling asleep | Poor sleep | Slightly poor | Normal |
| **Fatigue** | Frequent fatigue | Frequent inertia | Sometimes fatigue | Sometimes slightly fatigue | Not fatigued |
| **Pain** | Severe pain | Heavy pain | Moderate pain | Slight pain | Not painful |
| **Condition** | Bedridden | Most of the time in bed | Half the time in bed | Unable to work | Normal life and work |

Supplementary Table 3
