# Supplementary material for: Water‐filtered infrared A radiation hyperthermia combined with immunotherapy for advanced gastrointestinal tumours
Source: Cancer Med. 2024 Jul 24;13(14):e70024. doi: 10.1002/cam4.70024 (PMC11269209; doi:10.1002/cam4.70024)
Supplement: Supplementary file 9 — Table S4. [file CAM4-13-e70024-s008.docx]

| **Introduction of** **Hyperthermia Related Adverse Events (HRAEs)**  **Definition:** Subjective discomforts presents after hyperthermia and not present before hyperthermia, or considering related with hyperthermia but no other reason to explain. | | | |
| --- | --- | --- | --- |
|  | **Mild** | **Moderate** | **Severe** |
| **Acid reflux** | No need to stop hyperthermia. | Relieved after stopping hyperthermia. | Non-relieved after stopping hyperthermia. |
| **Breath lessness** | No need to increase oxygen flow. | Relieved after increasing oxygen flow. | Non-relieved after increasing oxygen flow. |
| **Chest discomfort** | Chest distress. | Chest pain. | With abnormal electrocardiogram. |
| **Cough** | Sporadic cough. | Paroxysmal cough. | Persistent cough. |
| **Dizziness** | Transient. | Non-persistence. | Persistence. |
| **Expectoration** | Disappeared after sputum expulsion. | Relieved after sputum expulsion. | Non-relieved after sputum expulsion. |
| **Headache** | NRS pain score: 1–3. | NRS pain score: 4–6. | NRS pain score: 7–10. |
| **Nausea** | Without Vomit. | With vomit. | Non-relieved after vomit. |
| **Palpitation** | Stable heart rate. | Heart rate increased and then stable. | Heart rate progressively increased. |
| **Thirst** | No need to drink water. | Relieved after drinking water. | Non-relieved after drinking water. |
| **Skin calor** | Relieved before the end of hyperthermia. | Relieved soon after hyperthermia. | Required medical intervention. |

Supplementary Table 4. Definition and criteria of hyperthermia related adverse events.
