# Supplementary material for: Water‐filtered infrared A radiation hyperthermia combined with immunotherapy for advanced gastrointestinal tumours
Source: Cancer Med. 2024 Jul 24;13(14):e70024. doi: 10.1002/cam4.70024 (PMC11269209; doi:10.1002/cam4.70024)
Supplement: Supplementary file 10 — Table S5. [file CAM4-13-e70024-s010.docx]

|  | Patients (n=18) |
| --- | --- |
| All grade irAEs | 4 (22.2%) |
| Ⅰ | 3 (16.7%) |
| Ⅱ | 1 (5.6%) |
| ≥Ⅲ | 0 (0.0%) |
| Grade Ⅰ | |
| Fever | 1 (5.6%) |
| Rash | 1 (5.6%) |
| Diarrhea | 1 (5.6%) |
| ALT elevation | 1 (5.6%) |
| Grade Ⅱ | |
| Pruritus | 1 (5.6%) |

Supplementary Table 5. The incidence of immune-related adverse events of the 18 patients
